# Conformational Variability of Amyloid-β and the Morphological Diversity of Its Aggregates †

**DOI:** 10.3390/molecules27154787

**Published:** 2022-07-26

**Authors:** Maho Yagi-Utsumi, Koichi Kato

**Affiliations:** 1 Graduate School of Pharmaceutical Sciences, Nagoya City University, Nagoya 467-8603, Japan; 2 Exploratory Research Center on Life and Living Systems and Institute for Molecular Science, National Institutes of Natural Sciences, Okazaki 444-8787, Japan

**Keywords:** aggregation, amyloid-β, cryo-electron microscopy, fibril, ganglioside, molecular chaperone, NMR spectroscopy

## Abstract

Protein folding is the most fundamental and universal example of biomolecular self-organization and is characterized as an intramolecular process. In contrast, amyloidogenic proteins can interact with one another, leading to protein aggregation. The energy landscape of amyloid fibril formation is characterized by many minima for different competing low-energy structures and, therefore, is much more enigmatic than that of multiple folding pathways. Thus, to understand the entire energy landscape of protein aggregation, it is important to elucidate the full picture of conformational changes and polymorphisms of amyloidogenic proteins. This review provides an overview of the conformational diversity of amyloid-β (Aβ) characterized from experimental and theoretical approaches. Aβ exhibits a high degree of conformational variability upon transiently interacting with various binding molecules in an unstructured conformation in a solution, forming an α-helical intermediate conformation on the membrane and undergoing a structural transition to the β-conformation of amyloid fibrils. This review also outlines the structural polymorphism of Aβ amyloid fibrils depending on environmental factors. A comprehensive understanding of the energy landscape of amyloid formation considering various environmental factors will promote drug discovery and therapeutic strategies by controlling the fibril formation pathway and targeting the consequent morphology of aggregated structures.

## 1. Introduction

Protein folding is the most fundamental and universal example of biomolecular self-organization and is characterized as an intramolecular process where nascent unfolded polypeptide chains assemble into their highly ordered native conformations [1]. In the protein folding process, the unfolded protein exhibits various conformations, passes through several folding intermediates, and descends on a potential free-energy surface toward a thermodynamically favorable native state. However, the process of correct protein folding can fail and polypeptide chains can fall into incorrect conformational states. These misfolded proteins can interact with one another, leading to protein aggregation [1,2,3,4]. In such misfolding, the amyloid fibrils are in one of the most stable thermodynamic states in the energy landscape [5]. Kinetically trapped misfolded intermediates are assumed to promote specific and nonspecific intermolecular interactions, thereby resulting in the assembly of various forms of aggregates such as oligomers, amorphous aggregates, and amyloid fibrils [5,6,7]. It is suggested that the energy landscape of amyloid fibril formation is characterized by a large number of minima for different competing low-energy structures [7,8,9] and, therefore, is much more enigmatic than that of multiple folding pathways. Furthermore, not only the final amyloid structures but also the aggregation processes are significantly altered by various environmental factors. Thus, to understand the entire energy landscape of protein aggregation, it is important to elucidate the full picture of conformational changes and polymorphisms of amyloidogenic proteins, which can depend on environmental factors.

Amyloid-β (Aβ) is one of the most extensively studied amyloidogenic proteins, primarily because of its pathological significance associated with Alzheimer’s disease (AD). Various experimental and theoretical approaches have been employed to characterize the structure and interactions of Aβ, revealing its conformational transformability. Intriguingly, the assembly of Aβ molecules is significantly promoted through an interaction with ganglioside GM1, which is a glycosphingolipid abundant in neuronal cell membranes [10,11,12]. In this review, we outline our current knowledge on the conformational transitions of Aβ, depending on the surrounding environments and binding molecules, highlighting its conformational variability and the morphological diversity of its aggregates.

## 2. Transient Interaction of Aβ in a Solution

Aβ is a product of the sequential cleavage of the type-I membrane glycoprotein, amyloid precursor protein (APP), by β- and γ-secretases [13]. The C-terminal region of Aβ is part of the transmembrane domain of APP and originally forms an α-helix conformation in the membrane [14]. After cleavage, the Aβ peptide dissociates from the membrane in the unstructured state and undergoes various structural changes [15]. Molecular dynamics (MD) simulations and nuclear magnetic resonance (NMR) spectroscopy have illustrated that monomeric Aβ conformation is mainly disordered in a solution and rapidly interconverts between many diverse conformational states [16]. Thus, it should be delineated as a conformational ensemble rather than a single dominant folded structure. The dynamic disordered state of Aβ is deeply involved in the process of its aggregation through transient Aβ–Aβ interactions. The dimerization and oligomerization processes of Aβ are well-characterized by MD simulations [17,18], indicating that the C-terminal regions of the Aβ molecules dominantly initiate their interactions.

The transient conformations of monomeric Aβ bound to large fibrils and oligomers have been indirectly observed by NMR techniques such as relaxation dispersion and saturation transfer experiments, which explored the invisible NMR states [19,20]. These data indicated that the central hydrophobic region of monomeric Aβ mainly mediates its interactions with the Aβ(1–40) oligomers whilst the C-terminal hydrophobic regions of both Aβ(1–40) and Aβ(1–42), along with their central hydrophobic regions, are involved in their interactions with the protofibril surface.

**Figure 1:**
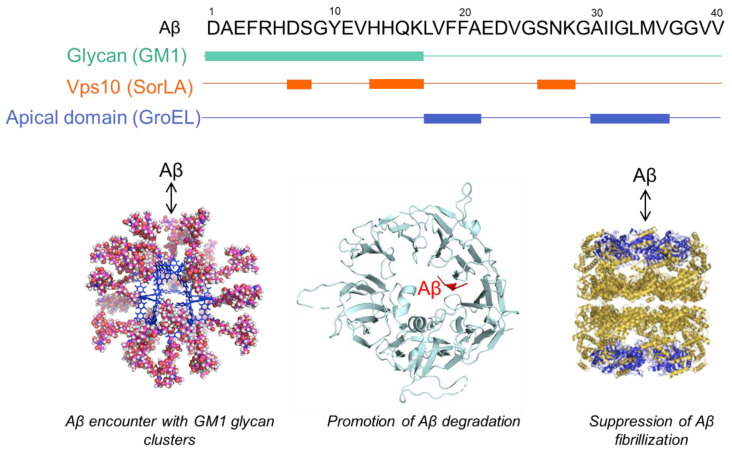
Transient interaction of Aβ with binding molecules. Each binding site on Aβ with the spherical complex displaying GM1 glycans (green), SorLA Vsp10 domain (orange), and apical domain of GroEL (blue) is represented with the primary structure of Aβ. The molecular graphics of GroEL and SorLA Vps10 domain with Aβ are based on PDB: 1KP8 and 3WSZ, respectively. The molecular graphics of the spherical complex displaying GM1 glycans are adopted with permission from reference [21]. 2015, WILEY–VCH Verlag GmbH & Co. KGaA, Weinheim, Germany.

Aβ exhibits essential conformational plasticity and adaptability during transient weak interactions with other proteins, lipids, and chemical compounds. For instance, Aβ can interact with the spherical complex, displaying pentasaccharide moieties derived from ganglioside GM1 and enabling the observation of transient glycan–protein interactions [21] (Figure 1). NMR data, along with MD simulations, have indicated that the N-terminal segment of Aβ(1–40), especially the hydrophilic His13-His14-Gln15 segment, is selectively involved in the interaction with the GM1 pentasaccharide cluster whereas the C-terminal segment is scarcely involved in the interaction [21,22]. It has been reported that α-synuclein (αSN), an intrinsically disordered protein involved in Parkinson’s disease, also forms weak encounter complexes with ganglioside-embedding small bicelles on an initial membrane-landing process of αSN [23]. Transient interactions of its N-terminal segment were observed for GM1 or GM2, but not GM3, which did not involve any secondary structure formation of αSN. In both Aβ and αSN, the initial encounters are mediated through their N-terminal *ganglioside-philic* segments without any secondary structure formation.

Furthermore, it has been reported that various molecular chaperones such as heat shock proteins, prefoldins, and small heat shock proteins can bind Aβ and thereby inhibit its aggregation and mediate Aβ degradation via the ubiquitin-proteasome system or autophagy [24]. Molecular chaperones assist with the folding of unstructured nascent polypeptide chains into their native conformational state mostly by preventing their off-pathway intermolecular interactions in the energy landscape [5,25]. GroEL, a member of the chaperonine family of molecular chaperons, can suppress Aβ(1–40) amyloid formation by transiently interacting with its two hydrophobic segments, Leu_17_-Ala_21_ and Ala_30_-Val_36_ of Aβ(1–40), which contain key residues in fibril formation [26] (Figure 1). Intriguingly, the specific hydrophobic segment of αSN is capable of interacting with the eukaryotic chaperone PDI [27], the bacterial chaperone GroEL [28], and the archaeal chaperone PbaB [29], suggesting that αSN displays a *chaperone-philic* binding motif that can be widely recognized as a mimic of misfolded protein hallmarks. NMR data also indicate that Aβ as well as αSN, when noncovalently tethered to GroEL, remain largely unfolded and highly mobile.

Such dynamic and loose complexes have also been observed for the neuronal sorting receptor SorLA, which captures Aβ inside a tunnel to extend the β-sheet of one of its propeller blades [30] (Figure 1). In conjunction with X-ray crystallography, NMR spectroscopy demonstrated that Aβ can remain attached to SorLA whilst undergoing transitions among different bound states involving multiple capture sequences, suggesting that SorLA binds Aβ monomers through weak interactions and escorts them to lysosomes for degradation.

## 3. Assembly of the Intermediate Structures of Aβ on Membranes

The aggregation and deposition of Aβ on neuronal cell membranes are deeply involved in the pathogenesis of AD. Aβ can exhibit a free three-dimensional motion in an aqueous solution whilst Aβ molecular motion is restricted at the two-dimensional membrane interface, thereby facilitating Aβ–Aβ interactions [12,31]. Therefore, to understand the molecular mechanisms of Aβ fibrillization, it is necessary to identify the effects of spatial limitations at the membrane interface on the molecular motions and intermolecular interactions of Aβ molecules.

**Figure 2:**
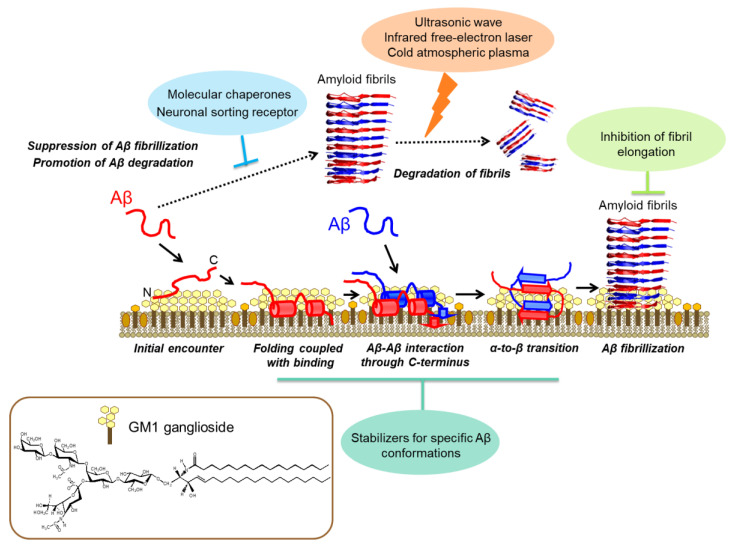
Schematic representation of the structural basis of the conformational transition and molecular assembly of Aβ promoted on GM1 ganglioside clusters on the neuronal cell membrane and the structure-based therapeutic strategies. After the initial encounter, the GM1 cluster captures Aβ at the hydrophobic/hydrophilic interface, which facilitates α-helix formation, thereby restricting the spatial rearrangements of Aβ molecules. Consequently, a specific intermolecular interaction between Aβ molecules is enhanced on the GM1 cluster, leading to their α-to-β conformational transition, resulting in amyloid fibril formation. Several proteins, including molecular chaperones, capture Aβ and thereby suppress its fibrillization. Irradiation with ultrasonic waves, an infrared free-electron laser, and cold atmospheric plasma can break down amyloid fibrils. Adapted with permission from reference [12]. 2019, The Pharmaceutical Society of Japan.

The ganglioside clusters are known to catalyze the self-assembly of amyloidogenic proteins such as Aβ, αSN, and prion protein through their interactions with gangliosides in a nonstoichiometric, but specific, manner [10,11,32,33,34]. Furthermore, amyloid fibrils on GM1-containing liposomes have been reported to be more toxic than those formed in an aqueous solution [35,36]. To provide structural insights into the conformational transition and molecular assembly of Aβ promoted in membrane environments, a series of NMR studies were carried out to characterize the interactions of Aβ with GM1 clusters by employing various membrane models [37,38,39,40,41,42,43] (Figure 2). The NMR data indicated that the GM1 clusters capture Aβ in an α-helical conformation at the hydrophobic/hydrophilic interfaces, restricting its spatial rearrangement: the two helical segments and the C-terminal portion of Aβ are in contact with the hydrophobic interior whilst leaving the remaining regions exposed to the hydrophilic environment of the GM1 cluster [42]. MD simulations have confirmed the topological mode of Aβ at the hydrophobic/hydrophilic interface [31]. The formation of the α-helical structure of Aβ has also been observed in membrane-mimicking micelles [44,45,46]. However, MD simulations have also indicated that Aβ α-helices are not stable and tend to form a β-hairpin structure because conformational entropy loss on the hairpin formation is smaller at the planar interface than in a free solution [31]. It is conceivable that such entropic effects, along with the higher local concentration of Aβ molecules at the hydrophobic/hydrophilic interface, facilitate their intermolecular interaction coupled with an α-to-β conformational transition on the ganglioside clusters, leading to amyloid fibril formation [31,40]. Indeed, Aβ bound to large, flat vesicles composed of 1,2-dimyristoyl-sn-glycero-3-phosphocholine forms a partially ordered conformation, in which only the C-terminal segments are involved in a parallel β-structure whilst leaving the N-terminal segment disordered [47]. Very recently, a nonfibrillar Aβ assemblage formed on a GM1-containing membrane was identified as a double-layered anti-parallel β-structure [43]. This unique assemblage itself was not transformed into fibrils, but rather provided a solvent-exposed hydrophobic surface that facilitated the conversion of monomeric Aβ into fibrils.

These findings suggest that the GM1 clusters offer a unique platform for binding coupled with the conformational transition of Aβ molecules, thereby restricting their spatial rearrangements to promote specific intermolecular interactions leading to cross-β-sheet formation (Figure 2). This raises a novel medicinal strategy to suppress β-structure formation by stabilizing the α-helical structure of Aβ on the ganglioside clusters. Indeed, it was reported that compounds such as N1-decanoyl-diethylenetriamine that bind and stabilize the α-helical state of Aβ attenuated fibril formation and consequent toxicity in a Drosophila model of AD [48]. On the other hand, hereditary mutations have a potential impact on these on-membrane molecular events [49] as exemplified by the Flemish-type mutation (A21G). This mutation disrupts the first α-helix identified in wild-type Aβ(1–40) bound to lyso-GM1 micelles, rendering the unstructured N-terminal segment tethered to the residual C-terminal helix [37]. Thus, the mutational effects on Aβ conformation depend on the surrounding environments.

## 4. Structural Polymorphism of Amyloid Fibrils

Increasing structural data provided by cryo-electron microscopy (cryo-EM) and solid-state NMR spectroscopy demonstrate that the morphology of amyloid fibrils is significantly affected by various solution conditions such as the protein concentration, ionic strength, pH, temperature, and pressure [50,51,52]. X-ray diffraction studies have shown that amyloid fibrils share similar structural features characterized by a cross-β spine: a double β-sheet with each sheet running parallel to the fibril axis [53]. At the mesoscopic level, however, amyloid fibrils formed under the same conditions show considerable morphological diversity [54,55]. These molecular polymorphisms are assumed to be derived from differences in the number, relative orientation, and internal substructure of the protofilaments. Recent simulation studies have shown that the sequence-specific conformational heterogeneities of monomer ensembles are crucially associated with their aggregation propensities and the fibril polymorphisms can be caused by changes in the population of fibril-like states in the monomeric structures [56,57].

**Figure 3:**
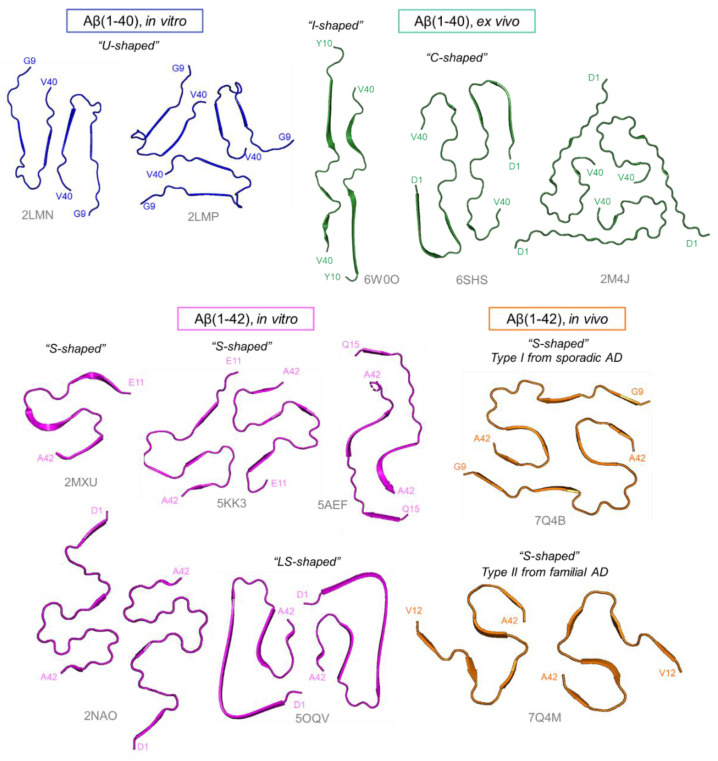
Aβ fibril structures solved by solid-state NMR and cryo-EM. The variety of fibril structures of Aβ(1–40) (blue, PDB: 2LMN, 2LMP) and Aβ(1–42) (magenta, PDB: 2MXU, 5KK3, 5AEF, 2NAO, 5OQV) fibrils prepared in vitro. Ex vivo, Aβ(1–40) seeded fibrils, which were formed by seed aggregation with recombinant Aβ(1–40) and ex vivo fibrils (green, PDB: 6W0O, 6SHS, 2M4J). The Aβ(1–42) fibrils were extracted from human AD brains (orange, PDB: 7Q4B, 7Q4M).

Solid-state NMR-derived high-resolution structural models have visualized that Aβ(1–42) fibrils adopt an S-shaped conformation [58,59,60,61] whereas Aβ(1–40) fibrils assume a U-shaped conformation [62,63] (Figure 3). Even in a U-shaped conformation, there is a variation in the interprotofilament interface in Aβ fibrils [64].

Recent breakthroughs in cryo-EM have yielded the atomic structures of Aβ filaments extracted from AD brains (Figure 3). The structures of the Aβ(1–42) filaments from human AD brains were identified by two types of S-shaped protofilament folds [65] whereas those of the filaments assembled in vitro had an overall LS-shaped topology of individual subunits in the cross-β structure [66]. In the case of Aβ(1–40) fibrils, high-resolution cryo-EM data identified the most prevalent polymorph for fibrils in typical AD patients as I-shaped protofilament folds [67]; another cryo-EM study determined C-shaped folds in brain-derived fibrils [68] where both the N- and C-terminal ends of Aβ were folded back onto the central peptide domain. These morphological differences suggest that Aβ fibrils may adopt disease-specific molecular conformers such as prion and tau strains, depending on the differences in individual brain environments [50,55,69]. Intriguingly, significant differences have also been found in the amyloid formation kinetics and fibril morphology between microgravity-grown and ground-grown Aβ(1–40) amyloids [70]. These data suggest that Aβ fibril formation on the ground is kinetically trapped in a metastable state whereas it proceeds more slowly through a thermodynamic control under microgravity conditions, resulting in the observed morphological differences in Aβ(1–40) fibrils.

The N-terminal regions adjacent to the fibril cores are often invisible or ambiguous in the solid-state NMR- and cryo-EM-derived structures of Aβ fibrils due to structural disorders and/or high mobility. Furthermore, it has been suggested that amyloid fibril cores themselves fluctuate and are heterogeneous, causing morphological diversity in one filament. An MD simulation based on the NMR-derived structural model of an Aβ(1–42) fibril indicated that the protomer at the growing end of an amyloid fibril adopts a β-hairpin conformation with less fluctuation compared with the flexible opposing terminal protomer [71]. These differences in the conformational fluctuation of the two ends of fibrils can explain the experimentally determined unidirectionality of fibril elongation [72,73].

It has been reported that Aβ fibrils can be broken down via irradiation with ultrasonic waves, an infrared free-electron laser, and cold atmospheric plasma by experimental and theoretical approaches [18,74] (Figure 2). Therefore, not only the suppression of the fibril formation but also the degradation of amyloid fibrils can be potential therapeutic strategies for neurodegenerative diseases in the future.

## 5. Conclusions

Aβ exhibits a high degree of conformational variability upon transiently interacting with binding molecules in an unstructured conformation in a solution, forming an α-helical intermediate conformation on the membrane and undergoing a structural transition to the β-conformation of amyloid fibrils. Despite the cumulative structural data, a comprehensive understanding of the molecular mechanisms behind amyloid polymorphisms remains largely unexplored as a variety of factors can influence the molecular assembly process. Recently, the accuracy of protein structure predictions based on deep learning methods has been dramatically improved and, in the case of natively folded proteins, their three-dimensional structures can now be reliably predicted from amino acid sequences [75,76]. However, it is currently difficult to accurately predict amyloid structures from amino acid sequences because amyloid fibrils are aggregates of many protomers that can form various polymorphic structures despite the same amino acid sequence [9]. Moreover, morphological diversity can be seen for amyloid fibrils grown in the same solution [70,77,78]. In addition, current machine learning methods are not yet capable of predicting protein folding and aggregation pathways.

For a detailed and integrated understanding of the energy landscape of protein aggregation, it is essential to characterize the structures of amyloid fibrils corresponding with the number of heterogeneous minima and to elucidate the amyloid formation processes, including the intermediate structures. Moreover, various environmental factors can significantly influence the amyloid structures and aggregation kinetics. Hence, a comprehensive understanding of the energy landscape of amyloid formation considering such environmental factors will promote drug discovery and therapeutic strategies by controlling the fibril formation pathway and targeting the consequent morphology of the aggregated structures. To address this issue, it is important to further accumulate high-quality data from experimental and computational approaches, to develop informatics-based methods for structure predictions, and to interpret these data from a physicochemical perspective.
